# From HAM-Nat to the “Physikum” – Analysis of the study success parameters before and after the introduction of a science test in the approval procedure

**DOI:** 10.3205/zma001176

**Published:** 2018-08-15

**Authors:** Katrin Werwick, Kirstin Winkler-Stuck, Bernt-Peter Robra

**Affiliations:** 1 Otto-von-Guericke University Magdeburg, Faculty of Medicine, Department for Student Affairs, Magdeburg, Germany; 2 Otto-von-Guericke University Magdeburg, Faculty of Medicine, Department of Social Medicine and Health Economics, Magdeburg, Germany

**Keywords:** Selection procedure, HAM-Nat, medical studies, AdH, selection of applicants for studying medicine, 1st section of the medical state exam

## Abstract

**Background/Objectives:** For the winter semester 2012/13, the Medical School of Otto-von-Guericke University Magdeburg introduced the HAM-Nat test (Hamburg Assessment Test for Medical Degrees - Natural Sciences Section) for the selection of its study applicants with the aim of improving the academic success of their students in the pre-clinical part which has a heavy emphasis on natural sciences. The study examines the extent to which the new University Selection Procedure (AdH) influences two criteria for measuring students’ success, compliance with the standard period of study up until the first part of the medical state exam (M1) and its result.

**Methodology: **A comparison of above-mentioned parameters for measuring student success for the matriculation years 2008-2011 (no HAM-Nat test, Pre-Matriculation) and those of the matriculation years 2012-2014 (Nat-Matriculation), whose students have passed the HAM-Nat test in the selection process of the university. In addition, it was taken into account the number of course certificates gained within the standard time period. In the Nat-Matriculation, the HAM-Nat results were merged with the associated M1 exam results.

**Results: **The proportion of AdH students who were admitted to the Physikum (first part of the medical state exam (M1)) within the standard period of study only increased slightly in the period studied. Within the AdH group, 70% of the Pre-Matriculation group gained entry to the second phase of studies without delay, rising to 78% in the AdH-group of the Nat-Matriculation. For all admission groups taken together, the overall grades for the first section of the medical state exam 2010-2016 show a positive trend, regardless of the selection procedure. The proportion of correctly answered questions in the nationwide M1 increased accordingly in the period studied.

The better those matriculating had performed in the HAM-Nat test, the better their results were in the written and oral parts of the first part of the medical state exam.

**Conclusion: **Although a significant proportion of students in the AdH group had obtained their place of study only on the basis of their test result and the score in the HAM-Nat test only weakly correlated with the school leaving grade (Abitur), the quantifiable study success parameters to date - in an albeit short observation period before and after introduction of the test - improved slightly. The number of Nat-Matriculations is too low to be able to assess the effect of the HAM-Nat test bearing in mind natural fluctuations. Nevertheless, the HAM-Nat test as an instrument of selection also made it possible for candidates with originally insufficient Abitur grades to gain admission without negative effects on the study success of the AdH cohort.

## Zusammenfassung

**Hintergrund/Zielsetzung: **Die Medizinische Fakultät der Otto-von-Guericke Universität Magdeburg hat zum Wintersemester 2012/13 den HAM-Nat-Test (Hamburger Auswahlverfahren für Medizinische Studiengänge – Naturwissenschaftsteil) zur Auswahl ihrer Studienbewerber mit dem Ziel eingeführt, den Studienerfolg ihrer Studierenden im vorrangig naturwissenschaftlich geprägten vorklinischen Studienabschnitt zu verbessern. Untersucht wird, inwieweit das neue Auswahlverfahren der Hochschule (AdH) zwei Kriterien des Studienerfolgs beeinflusst, nämlich die Einhaltung der Regelstudienzeit bis zum 1. Abschnitt der Ärztlichen Prüfung (M1) und dessen Ergebnis.

**Methodik: **Die genannten Studienerfolgsparameter der Matrikel 2008-2011 (ohne HAM-Nat-Test, Vor-Matrikel) werden mit denen der Matrikel 2012-2014 (Nat-Matrikel) verglichen, deren Studierende im Auswahlverfahren der Hochschule den HAM-Nat-Test abgelegt haben. Außerdem wurde die Anzahl der in der Regelzeit bestandenen Leistungsnachweise berücksichtigt. In der Nat-Matrikel wurden die HAM-Nat-Ergebnisse mit den zugehörigen M1-Prüfungsergebnissen zusammengeführt.

**Ergebnisse: **Der Anteil der AdH-Studierenden, der in der Regelstudienzeit zur M1-Prüfung zugelassen werden konnte, ist in der untersuchten Zeitreihe nur gering gestiegen. Innerhalb der AdH Gruppe erreichten 70% der Vor-Matrikel verzögerungsfrei den zweiten Studienabschnitt, in der AdH-Gruppe der Nat-Matrikel 78%. Für alle Zulassungsquoten zusammengenommen ist bzgl. der Gesamtprädikate im 1. Abschnitt der Ärztlichen Prüfung von 2010-2016 unabhängig vom Auswahlverfahren ein positiver Trend zu verzeichnen. Der Anteil der richtig beantworteten Fragen in der bundesweiten M1-Prüfung stieg dazu passend im Beobachtungszeitraum.

Je besser die Immatrikulierten im HAM-Nat-Test abgeschnitten hatten, desto besser waren ihre Ergebnisse im schriftlichen und im mündlichen Teil des 1. Abschnitts der ärztlichen Prüfung.

**Schlussfolgerung:** Obwohl ein nennenswerter Teil der Studierenden in der AdH-Quote ihren Studienplatz erst aufgrund ihres Testergebnisses erhalten hatte und die Punktzahl im HAM-Nat-Test nur schwach mit der Abiturnote korreliert, haben sich die bisher quantifizierbaren Studienerfolgsparameter im allerdings nur kurzen Beobachtungszeitraum vor und nach Einführung des Tests gering positiv verändert. Die Zahl der Nat-Matrikel ist zu gering, um den Effekt des HAM-Nat-Tests in seinen natürlichen Schwankungen beurteilen zu können. Dennoch ermöglicht der HAM-Nat-Test als Auswahlinstrument auch Bewerbern mit primär unzureichend zulassungsfähigen Abiturdurchschnittsnoten Erfolg im Zulassungsverfahren, ohne den Studienerfolg der AdH-Kohorte zu verschlechtern.

## Background

Using their own selection criteria (AdH) German medical schools can award 60% of the university places that are available after deduction of preliminary quotas, plus any additional places (for example from the quote for candidates with the highest Abitur grades which have not been taken up) which have been redistributed for allocation by the university. The selection procedure is primarily performance based [http://www.landesrecht.sachsen-anhalt.de/jportal/;jsessionid=2A69786A56277F588FA9F903070C8FCE.jp14?quelle=jlink&query=HSchulZulG+ST&psml=bssahprod.psml&max=true&aiz=true#jlr-HSchulZulGST2012rahmen]. In addition to the Abitur^1^ grade (“Level of Qualification”), individual grades of the Abitur report card and/or

1. the result of a subject-specific academic ability test,
2. the type of vocational training or professional activity and/or
3. the result of a selection interview can be taken into account in the selection.

The possible selection instruments differ in terms of the residual dominance of the Abitur grade as a performance criterion and in their necessary logistics. Most medical schools no longer look for applicants by Abitur grade alone but consider other selection criteria [1]. In a longitudinal analysis Kadmon et al. [2] have shown that the Abitur grade is a “predictor of academic performance”. Nevertheless, the significance of the Abitur grade for the success of studying in the clinical phase of studies and for professional success has not been sufficiently researched [3]. In addition to considering professional experience in the medical field, some faculties conduct structured interviews. Göttingen, for example, carries out very complex and highly structured “Multiple Mini-Interviews” (MMI) [4]. The procedure consists of a structured interview and a practical test (wards with simulated patients) and is suitable for predicting outcome criteria in social and communicative domains. Hamburg and Berlin, like Magdeburg, carry out the HAM-Nat test [4], [5], [6], [7], [8], [9], [10]. Numerous other medical faculties take into account the Test for Medical Studies (TMS) results in the selection of their applicants [1]. Students with poor Abitur grades but who passed the TMS achieved better study results in the first phase of studies than the top Abitur students who only achieved moderate TMS results [11]. Communicative and social competence, empathy and study motivation are not tested by the HAM-Nat test or TMS. None of these criteria are prospectively validated with regard to professional success [4], [3]. 

In the “Master Plan for Medical Studies 2020” [https://www.bmbf.de/de/masterplan-medizinstudium-2020-4024.html, as of 11.04.17] at least two additional selection criteria are called for in addition to the Abitur grade. According to a proposal of the MFT (Medical School Association) and bvmd (Federal Representation of Medical Students in Germany) a nationwide uniform test ought to be included, for example TMS or HAM-Nat (Hamburg Science Test). This would grant selection tests a particular importance in the future. It therefore makes sense to investigate the impact of current tests. In addition, in future the selection of applicants will place more emphasis on practical and social and communication skills as well as qualifications already acquired in medical professions (see Federal Constitutional Court, Judgment of the First Chamber, December 19, 2017 – 1 BvL 3/14 – Rn. (1-253)). The Federal Constitutional Court considers it unconstitutional if, in addition to the average Abitur grade, “no further selection criteria are taken into account with due weight in the universities’ selection procedure. However, given that the criteria as a whole must guarantee a sufficient predictive capacity for the aptitude profile, further longitudinal studies on the outcome of the selection process are important.” 

Until the winter semester 2011/12 the Abitur grade was the sole selection criterion in the selection procedure of the Medical School of Magdeburg. For the winter semester 2012/13, the faculty introduced a science test, namely the HAM-Nat test, for the selection of its study applicants. This had been specially developed and validated at the Medical School of University Hamburg for the selection of medical students and has since been the subject of further evaluation [5], [6], [7], [8], [9]. It is a multiple-choice test with questions on medically relevant aspects of biology, physics, chemistry and mathematics. As part of the selection procedure, first the 700 applicants with the highest University Entrance Qualification (HZB) grades, who have given Magdeburg as their first preference, are ranked according to their average HZB grade. The top 25 applicants will be directly admitted to study, i.e. without taking further tests (excellence quota). From 26 onwards, places will be awarded according to their average Abitur grade in combination with the result of the selection test. The average grade of a candidate’s university entrance qualification is converted into a score of 60 (1.0, the top Abitur grade) to 0 (above grade 4.0) using a linear scale. For the test result of the HAM-Nat test up to 59 points are awarded (59 x number of correctly answered questions/number of scored questions). The rank of an applicant from place 26 onwards in the AdH procedure is determined through the sum of their two scores. A higher total score means a better ranking (see [10] for details). 

The present compilation compares the study results, in particular the results in the first section of the medical state exam of the Pre-Matriculation group with those of the Nat-Matriculation group. In addition, the Abitur grades and the HAM-Nat test results of the Nat-Matriculation group are correlated with their results in the first section of the medical state exam. In addition, the number of course credits gained in the standard time period was taken into account in the AdH rate for all admission cohorts. 

## Methods

The matriculation years 2008-2011 (without HAM-Nat; n=790) and 2012-2014 (with Ham-Nat; n=578), hereafter referred to as Pre-Matriculation and Nat-Matriculation groups, were assessed in terms of compliance with the standard period of study up to the first part of the medical state exam (M1), their M1 results and the number of pre-clinical course credits gained in the standard time period (see Table 1 (Tab. 1)). In the analysis, the students of all admission groups are included, the results of the students from the AdH are presented in detail and compared with cohorts who gained admission through waiting time or top Abitur grades. 

Since the Medical Licensure Act does not stipulate grades for course certificates gained (except for the optional subject) in the first phase of studies, the number of course credits gained within the standard time period according to Annex 1 of the Medical Licensure Act was used for a comparison of study achievements. In addition, the 2012-2014 HAM-Nat results were combined with the associated M1 exam results and also compared with the nationwide M1 exam results.

All data were anonymized for analysis and evaluated with the statistics program SPSS (Version 24). Correlation analyses of the Nat-points with the Abitur grades and with the individual M1 exam results (written, oral, total) of the Nat-Matriculation group and regression analyses were carried out of the state exam grades (dependent variable) with the HAM-Nat result and the Abitur grade (independent characteristics). The Pre-Matriculation group was examined for the influence of the Abitur grade on the M1 result. Frequency differences between groups were evaluated for significance using Fisher’s Exact Test, mean differences using t-Test and the Mann-Whitney-U-Test for significance (error probability p≤0.05 without adjustment for multiple testing). 

## Results

### Comparison of the results of first section of the medical state exam 2010-2016 

In 2012 56%, in 2013 65% and in 2014 66% of the AdH group were admitted based on their test results (Paternoster effect [10]). The HAM-Nat test result is therefore very effective in terms of gaining admission. Table 1 (Tab. 1) compares the course of studies of the Pre-Matriculation group with the Nat-Matriculation group. 

In total, 66% of the students in the Pre-Matriculation group were admitted to the M1 exam within the standard period of study. Of these, 88% passed the exam at the first attempt. In total, 58% of those admitted usually reached the second stage of study within the standard time period. 

In total, 66% of the students in the Nat-Matriculation group were admitted to the M1 exam within the standard period of study. Of these, 96% passed the exam, so a total of 63% were able to progress to the next phase of study, 5 percentage points more than in the Pre-Matriculation group. However, considering the last percentages separately for each of the seven years, two of the years compared with and without test had the same overall success rates of 60% (without test 60%, 55%, 56%, 60%, with test 60%, 70%, 60%).

If only the students enrolled via the AdH are considered, then the proportion of those admitted within the standard time period to M1 is 75% and 79% respectively, proportionately more than in the entire matriculation, and there are also proportionately more students who reach the second phase of study within the standard time period than in the entire matriculation (70% and 78% respectively). However, the latter figure fluctuates from year to year: without test 73%, 60%, 60%, 87% and with test 82%, 87%, 64%. Thus the new selection procedure is not consistently more productive with regard to completing the M1 exam within the standard period of study. Of those matriculated via the top Abitur grade quota, 83% of the Pre-Matriculation group and 52% of the Nat-Matriculation group reach the second phase of study within the standard time period. All students (100%) admitted via the top Abitur grade quota who registered for the M1 exam in regular time passed the exam. Of those who gained admission via waiting time, only 37% of the Pre-Matriculation group and 28% of the Nat-Matriculation group continued to study having passed M1 in regular time.

If one compares the results of the first state exam in Magdeburg with the national average – for comparability only the reference group is taken into account for the standard period of study – in both periods compared, Magdeburg^2^ students answered more questions correctly than the national average. With an additional 3.5 correctly answered questions, the Pre-Matriculation group lay 1.4% above the national average, the Nat-Matriculation with additional 5.3 questions, 2.0%. The difference between the two periods is therefore very small, with a gain of 0.6 percentage points.

As expected, the grades of the AdH group in the first section of the medical state exam (M1) are consistently better than those of students across all admissions quota groups. This was already the case before the HAM-Nat test (see Table 2 (Tab. 2)) and did not change after the introduction of the HAM-Nat selection test. On the other hand, if one compares the average Abitur grades of those admitted via the AdH quota, the average of the Pre-Matriculation group was 1.36 and that of the Nat-Matriculation 1.64. So the AdH group, having completed the HAM-Nat test, on average had worse Abitur grades, but performed just as well or better in the M1 exam than the AdH group of the Pre-Matriculation group. 

If one summarizes the annual results (see Table 2 (Tab. 2)), the M1 state exam result of the whole intake improves from 2.8 (Pre-Matriculation) to 2.5 (Nat-Matriculation). The M1 average scores in the top Abitur grade group as well as in the Waiting Time group change only minimally. The M1 state exam result of the AdH quota group improves after the introduction of the test from 2.6 (Pre-Matriculation) to 2.4 (Nat-Matriculation). Thus, the state exam results in the AdH cohort improve slightly. Contributing to this are the comparatively unfavorable results of the Excellence Group (2.4), which did not have to pass a science exam to gain admission. 

#### Comparison of AdH students in the years 2008-2011 and 2012-2014 who exceeded the standard period of study 

The percentage of students from the AdH quota who were not admitted to the M1 state exam did not significantly decrease as a result of the introduction of the HAM-Nat test (see Table 1 (Tab. 1)). The course of study of these groups was further characterized by the number of missing course certificates. On average these students lack 2-3 course certificates for admission to the M1 exam, especially in chemistry, biochemistry, anatomy and physiology.

Students who have not completed 3 or more course certificates are examined in more detail. In the Pre-Matriculation group a total of 55% of those not admitted in time were missing 3 or more course certificates (116/210). In the AdH quota group the rate was 39% (46/117). Of the students in the Nat-Matriculation group who did not qualify for the M1 within the standard period of study, 41% were missing 3 or more course certificates (82/198). For those in the AdH quota group it was 33% (28/85). 

Thus in general, fewer students in the Nat-Matriculation group lack 3 or more course certificates for M1 admission compared to the Pre-Matriculation group. Within the AdH quota group, the effect is less pronounced. 

#### Relationship between HAM-Nat test results and state exam grades

The following applies to the Nat-Matriculation group: The more questions were answered correctly in the HAM-Nat test, the better the grades in the written part and the overall grades in the first part of the medical state exam (negative sign: candidates with high test scores have good M1 exam results) (see Table 3 (Tab. 3)). However, the correlation coefficients are only small. The HAM-Nat result also correlates weakly with the Abitur grades, although the internal consistency of the HAM-Nat test is almost the same in all three test years (Cronbach’s α 0.87 to 0.89). On average, the students who passed the HAM-Nat test in the AdH quota group were better in the M1 exam than the students who did not pass the test (Excellence Quota). The regression analysis shows a significant prediction of the written M1 exam results through the Nat score (p≤0.001). Together with the Nat score, there is no significant contribution from the Abitur grade. The Abitur grade alone is not a predictor of the M1 exam results. The variance component explained in the model is low at 5%. If the gender and the place HZB was gained (German Federal State/EU) were included as factors, this did not have any significant effects. Even when examining the Pre-Matriculation group on its own, there is no significant correlation between the Abitur grade and the result of the written M1 exam. 

## Discussion

The present study examines the introduction of the natural science test HAM-Nat in a before-and-after comparison at university location. In the group of university applicants who have gained admission via the AdH, the test has a high impact on gaining admission (Paternoster Effect). The HAM-Nat result correlates only weakly with the final Abitur grade, so compared with the previous situation where the final Abitur grade was the only criterion, the test adds additional information to the selection process. The study by Hampe et al. [4] shows a low correlation of the Nat result with the Abitur grade in Hamburg.

The first possible success indicator in the course of study is the proportion of those admitted to the M1 within the standard period of study. It is of the same size before and after the HAM Nat test. However, the proportion of students who pass this exam is slightly larger in the Nat-Matriculation group, so that in the three years with the HAM-Nat test, more students than before were able to proceed to the second phase of studies. This could be due to the fact that students who have been admitted via the HAM-Nat test have better knowledge in physics, biology and chemistry and thus can concentrate more on other subjects of the pre-clinical phase of studies. 

In the subgroup of AdH admissions, where the effects of the HAM-Nat test ought to be more evident than in the entire intake, the proportion of students admitted to the M1 exam within the standard time period increased from 75% to 79% and the proportion who successfully moved into the second phase of studies from 70% to 78% – despite an increasing number of students with an Abitur from other EU countries (German vs non-German Abitur). In both reporting periods, however, there are clear fluctuations between the results of the individual years, which are not explained by the available data. This relativizes the observed trend. The size of Nat-Matriculations group is too small to be able to judge the natural fluctuations of the HAM-Nat effect. 

The regression analysis has shown that the HAM-Nat score, but not the Abitur score, has a significant correlation with the result of the M1 exam. The Abitur grade alone is not a predictor of the M1 exam results. A contributing factor could be that the Abitur grades of those matriculated through the AdH show little variance. As Hampe et al. [4] found, the Abitur grades in different German federal states vary greatly, something that is likely to weaken their association with the result of a nationwide state exam. 

One limitation is that it is not certain that the exams sat in Magdeburg were of comparable difficulty in all years. An equal proportion of students who passed the M1 after four semesters (66%) in both compared groups speaks for similarly effective filters. Throughout the reporting period, there has been a constant effort to improve teaching but no reorganization of the curriculum. 

It is noticeable that in the Nat-Matriculation group 52% of the top Abitur achievers do not stay within the standard time period and also that the students of the Excellence Quota, who did not have to take a test, are less successful than the rest of the AdH group who sat the test. In the Waiting Time quota group, which does not take a test, only about a third (Pre-Matriculation – 37% and Nat-Matriculation – 28%) generally continue to study after the M1 exam.

Without a very high overhead (individual evaluations of the written exam and certificate achievements), due to course certificates in the first phase of studies being ungraded, no differentiated analysis of the connection between the Abitur grade, Advanced Courses (Leistungskurs) taken in the last 2 years of secondary school, the HAM-Nat result and subject-specific course achievements is possible. These relationships could only be further examined from the second phase of studies on, in which numerous grades and thus quantified evaluations are awarded. Also, a comparison between the medical faculties of Hamburg and Berlin, which also uses the HAM-Nat test to select applicants, can only be attempted towards the final phase of studies. This is because both locations are not involved in the first state exam of the IMPP, because they have established model courses. However, their curricula also differ from the standard course in Magdeburg in other respects. 

The HAM-Nat test also provides good opportunities for university applicants with an Abitur grade worse than 1.5 to gain admission to medical faculties, to get a good start in their studies and to get over the hurdle of the M1. However, the analysis does not allow a prognosis on the quality of the graduates regarding all facets of medical practice and professional success. Also, the HAM-Nat does not test social and communication skills. Since interviews are very time-consuming and need to be structured to ensure validity, the HAM-Nat test is an objective procedure that is a pragmatic approach for a small faculty. Tested scientific knowledge at the beginning of studies may be a necessary, but not necessarily a sufficient prerequisite for a successful medical career. It must be considered how in future social and communicative competences in Magdeburg can be considered in the selection process.

## Notes

^1^ including European Abitur equivalents

^2^ https://www.impp.de/internet/de/loesungen-und-ergebnisse.html, as at 18.04.17. The indication of the participants of the reference group Magdeburg at the IMPP may differ from the information provided by the STEX participants (see Table 1 (Tab. 1)). University changers are not included in Table 1 (Tab. 1). 

## Acknowledgements

The authors wish to offer special thanks to the consulted and consulting partners, in particular

- the members of the Admissions Committee,
- Prof. Dr. W. Hampe, University Medical Center Hamburg-Eppendorf, Center for Experimental Medicine, Institute of Biochemistry and Molecular Cell Biology
- Dr. F. W. Röhl, Institute of Biometry and Medical Informatics
- as well as the study participants.

## Competing interests

The authors declare that they have no competing interests. 

## Figures and Tables

**Table 1:**
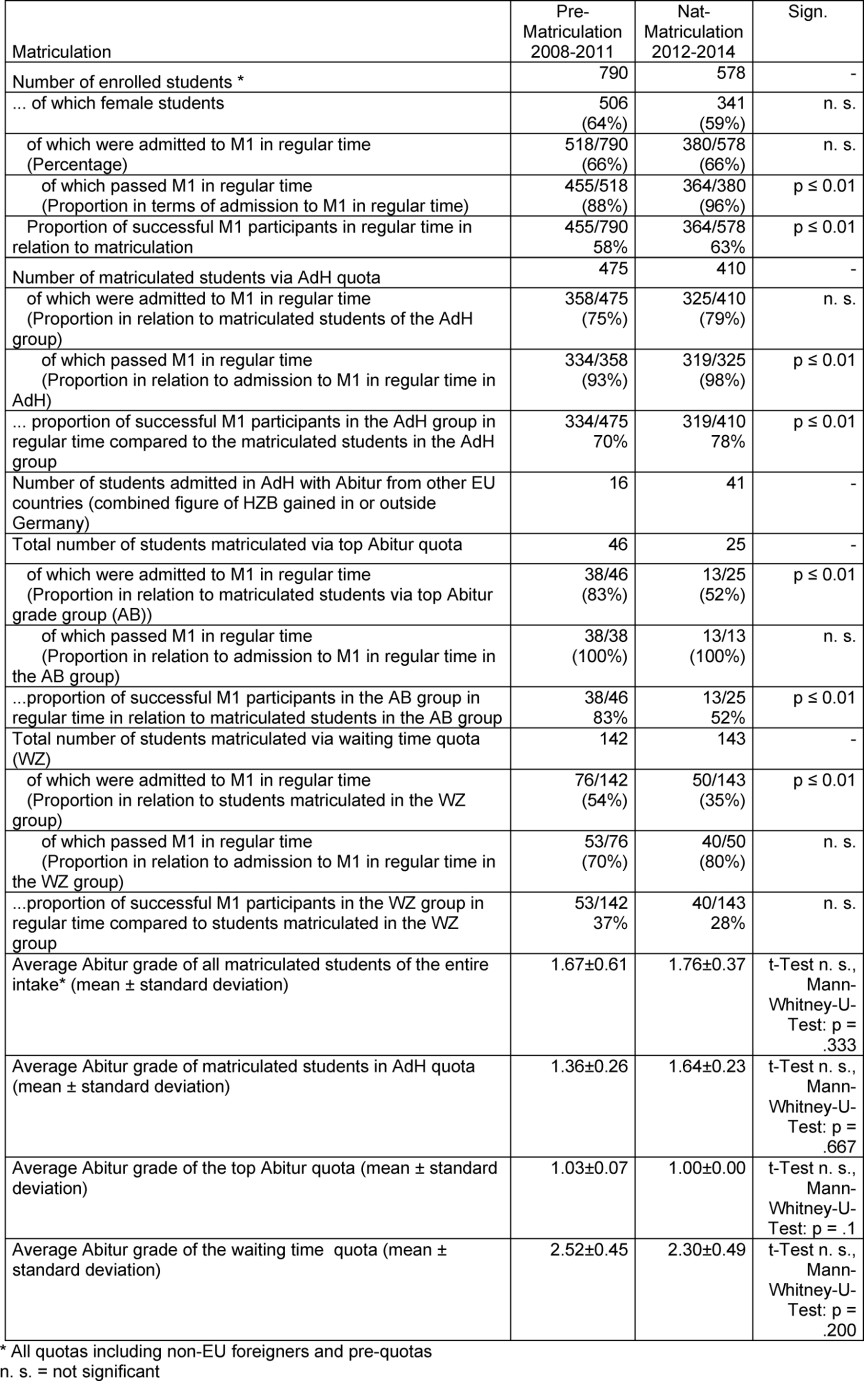
Course of studies of the Pre-Matriculation and Nat-Matriculation groups period up to M1

**Table 2:**
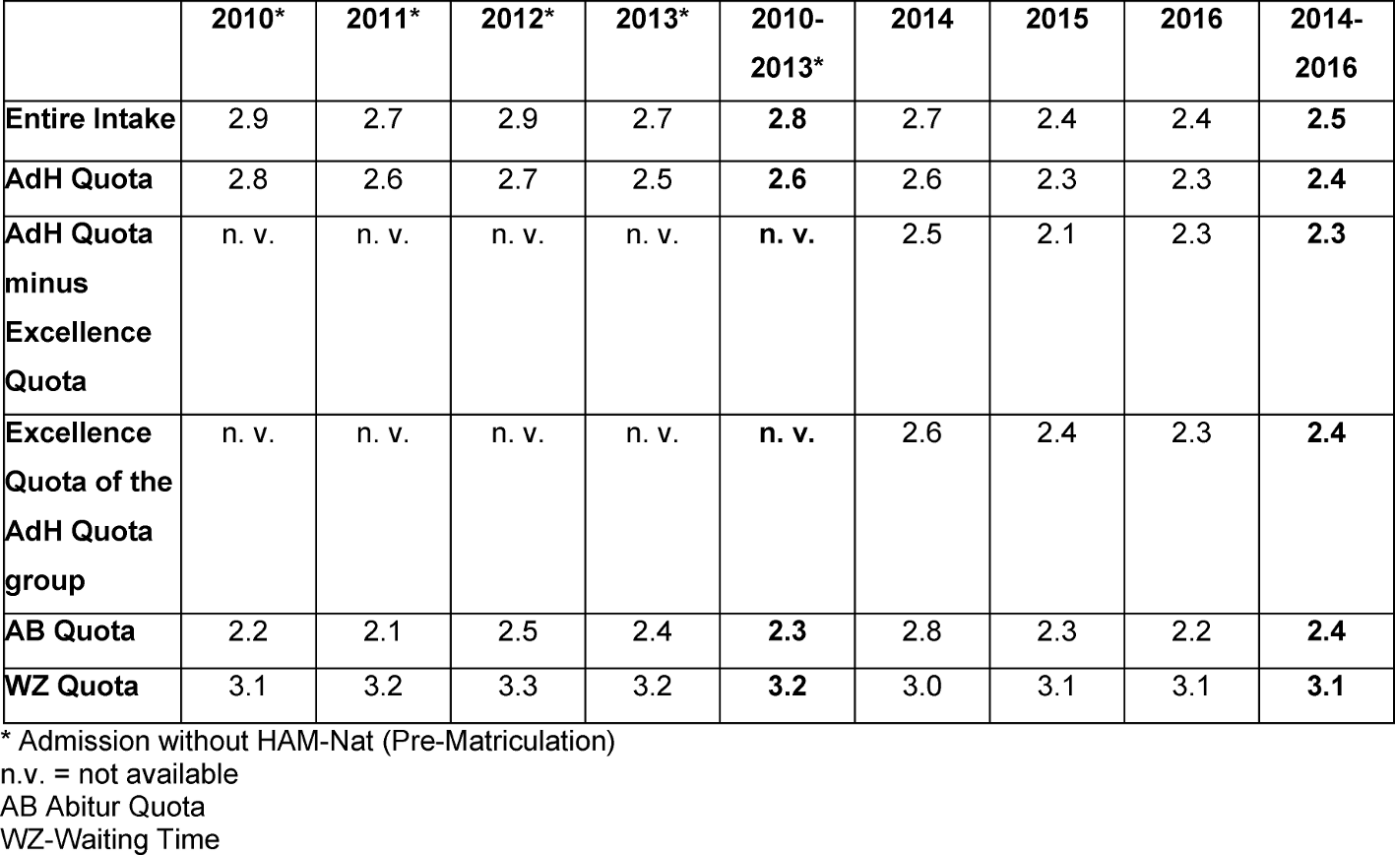
Mean values of the exam grades in the first section of the medical state exam, general and various subgroups, Magdeburg 2010-2016

**Table 3:**
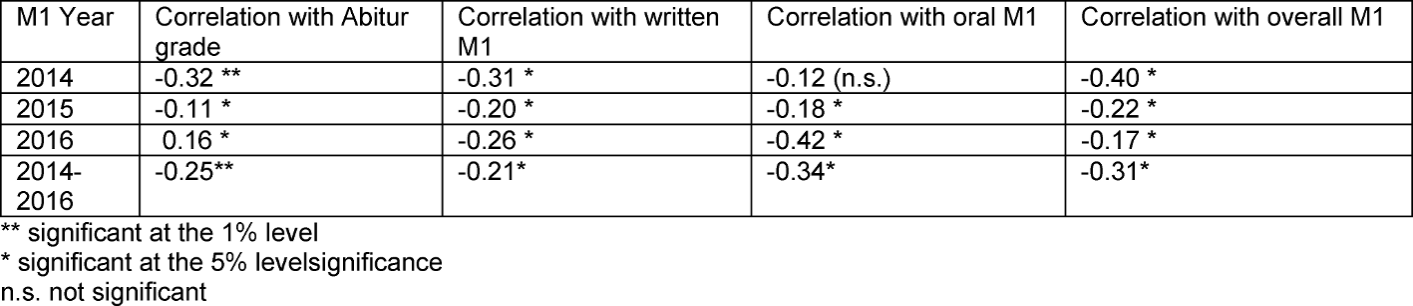
Correlation (r) of the Nat score with the Abitur grades and the M1 test results 2014 - 2016
